# Nanoparticles for Photodynamic Therapy of Breast Cancer: A Review of Recent Studies

**DOI:** 10.3390/molecules30071571

**Published:** 2025-03-31

**Authors:** Dorota Bartusik-Aebisher, Agnieszka Przygórzewska, Paweł Woźnicki, David Aebisher

**Affiliations:** 1 Department of Biochemistry and General Chemistry, Faculty of Medicine, Collegium Medicum, University of Rzeszów, 35-310 Rzeszów, Poland; dbartusikaebisher@ur.edu.pl; 2 English Division Science Club, Faculty of Medicine, Collegium Medicum, University of Rzeszów, 35-310 Rzeszów, Poland; ap117623@stud.ur.edu.pl; 3 Doctoral School, Faculty of Medicine, Collegium Medicum, University of Rzeszów, 35-310 Rzeszów, Poland; pawelw@dokt.ur.edu.pl; 4 Department of Photomedicine and Physical Chemistry, Faculty of Medicine, Collegium Medicum, University of Rzeszów, 35-310 Rzeszów, Poland

**Keywords:** nanoparticles, photodynamic therapy, PDT, latest research, breast cancer

## Abstract

Photodynamic therapy (PDT) is a therapeutic method based on the interaction between light and a photosensitizer. Supported by nanoparticles, this method represents a promising interdisciplinary approach for the treatment of many diseases. This article reviews the latest 2024 developments in the design and applications of nanoparticles dedicated to stand-alone PDT of breast cancer. Strategies to improve therapeutic efficacy by enhancing reactive oxygen species (ROS) production, precise delivery of photosensitizers and their stabilization in the systemic circulation are discussed, among others. Results from preclinical studies indicate significant improvements in therapeutic efficacy, including inhibition of tumor growth, reduction in metastasis and improvement of the immune microenvironment. The potential of these technologies to expand PDT applications in medicine and the need for further clinical trials to confirm their safety and efficacy are highlighted.

## 1. Introduction

Breast cancer represents a significant global health challenge, being the most frequently diagnosed malignant tumor worldwide. Approximately 2.26 million new cases of the disease were reported in 2020, making it the most common cancer in the general population and the leading cause of cancer deaths among women [1,2]. Breast cancer primarily affects women, and its incidence increases with age, with more than 80% of cases diagnosed in women over the age of 50 [3]. Epidemiological trends indicate a gradual increase in incidence of about 1% per year between 2012 and 2021, especially for localized-stage cancers and those with hormone receptor expression [4]. Pathologically, breast cancer is mainly classified as invasive ductal carcinoma (70–75%) and invasive lobular carcinoma (12–15%), according to the World Health Organization (WHO) classification. In addition, there are 18 rare histological subtypes, which together account for 0.5–5% of all cases [5]. In order to optimize therapeutic decisions and predict the course of the disease, breast cancer is also classified based on the expression of estrogen receptors (ER), progesterone receptors (PR) and HER2 protein. There are three main biological groups: HR-positive/HER2-negative (HR+/HER2-, ~70%), HER2-positive (HER2+, ~15–20%) and triple-negative breast cancer (TNBC, HR-/HER2-, ~15%) [6,7]. In clinical practice, an expanded molecular classification is used, based on immunohistochemical results, distinguishing four subtypes: luminal A, luminal B, HER2-positive and triple-negative [8]. With the increasing incidence of breast cancer and the diversity of its subtypes, the search for innovative and effective therapeutic approaches is becoming crucial. One promising therapeutic strategy may be photodynamic therapy (PDT).

PDT is an advanced therapeutic method based on the precise interaction between light and photosensitizer. The procedure involves the local or systemic administration of a photosensitizer, a photosensitive compound that selectively accumulates in the affected tissues. The key step in the therapy is the activation of the photosensitizer by absorbing light at a wavelength corresponding to the optimum absorbance of the photosensitizer, which initiates a cascade of photochemical reactions. This process leads to the generation of reactive oxygen species (ROS), which have a cytotoxic effect, destroying target pathological cells in a controlled manner and with minimal impact on surrounding healthy tissues [9]. Currently, this method has been approved for the treatment of head and neck cancer, esophageal cancer, pancreatic cancer, prostate cancer and esophageal squamous cell carcinoma. In addition, it is widely used in dermatology to target precancerous and cancerous lesions [10,11]. Work to optimize the efficacy of PDT is continually progressing [12], and one of the most promising developments is nanoparticles [13,14,15,16]. Nanoparticles are materials with sizes in the range of 1 to 100 nm [17] that show a wide range of medical applications, from molecular diagnostics to novel therapeutic approaches [18,19]. They represent an effective tool to overcome the limitations associated with traditional photosensitizers used in PDT, such as hydrophobicity, short circulation time in the blood after intravenous administration and, consequently, insufficient accumulation, retention and internalization in tumor tissues [20]. In addition, multifunctional nanomaterials can significantly increase the levels of reactive oxygen species in tumor tissues through mediation of photocatalytic oxygen production and Fenton reactions. The use of nanoparticles also makes it possible to improve light delivery to tumor tissues by being able to convert near-infrared (NIR) light, which penetrates deeper, into visible light or by using durable luminescent nanoparticles [21]. In addition to enhancing photosensitizer efficacy through physicochemically optimized passive targeting, active targeting using ligand modification and controlled stimulus-responsive release, nanomaterials offer the potential to combine PDT with other therapeutic strategies. Examples include chemotherapy, gene therapy, immunotherapy, photothermal therapy, hyperthermia and magnetothermal therapy, radiotherapy, or sonodynamic therapy, thus overcoming the limitations of traditional treatments [22]. In our narrative review, we summarize research on nanoparticles dedicated to PDT monotherapy of breast cancer published in the year 2024. An article search was conducted using the PubMed/MEDLINE database on 5 March 2025 using the phrase “PDT AND nanoparticles”. The number of 3772 articles was determined. The inclusion and exclusion criteria of the retrieved articles for this review are presented in Table 1. Finally, 13 original research articles describing nanoparticles for photodynamic therapy of breast cancer were eligible for inclusion.

## 2. Nanoparticles for Photodynamic Therapy of Breast Cancer

**Figure 1:**
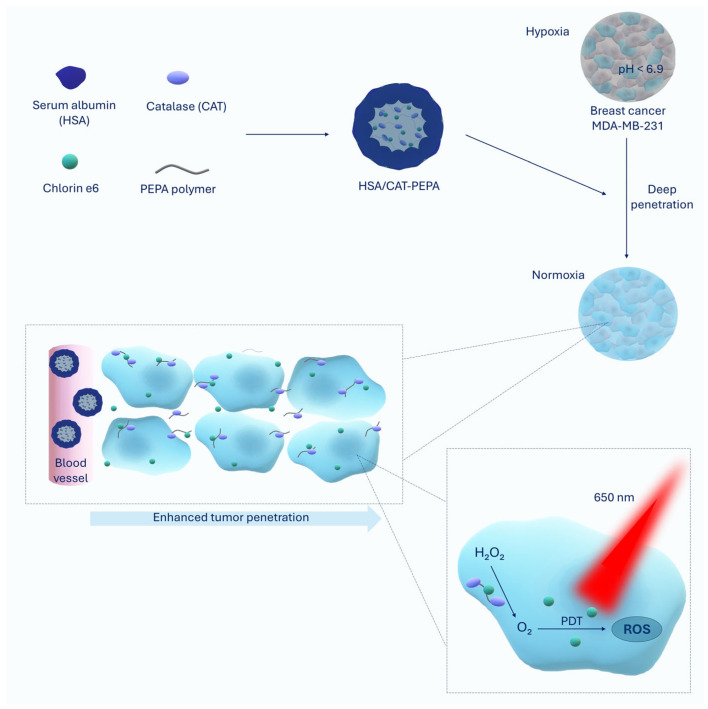
This figure shows nanoparticles based on a protein–polymer conjugate (HSA/CAT-PEPA) containing catalase (CAT), PEPA polymer, human serum albumin (HSA) and the photosensitizer Chlorin e6 (Ce6). In in vivo studies, HSA/CAT-PEPA nanoparticles were injected into mouse MDA-MB-231 human breast cancer cells. After intravenous administration, the nanoparticles effectively accumulated in the tumor, which is characterized by an acidic microenvironment (pH < 6.9) and hypoxia factors that significantly limit the efficacy of classical PDT. In response to low pH, the nanoparticles undergo controlled disintegration, leading to the release of catalase and Ce6, which facilitates deep penetration of the therapeutic components deep into the tumor tissue. The released catalase catalyzes the breakdown of hydrogen peroxide (H_2_O_2_) present in the tumor microenvironment into molecular oxygen (O_2_) and water, effectively eliminating hypoxia-one of the main limitations to PDT efficacy. Increased oxygen availability significantly improves the efficiency of ROS generation during the photodynamic process. Released Ce6, upon absorption of 650 nm light, enters an excited state, which initiates the photodynamic reaction, leading to the generation of ROS. The resulting ROS induce oxidative stress, causing damage to cell membranes, proteins and DNA of tumor cells, ultimately leading to their apoptosis and tumor elimination [23].

Huang et al. developed nanoparticles called HSA/CAT-PEPA to address hypoxia in the tumor microenvironment [23]. Rapid and uncontrolled proliferation of tumor cells limits oxygen availability, making inadequate blood supply or hypoxia a typical feature of the tumor microenvironment in almost all solid tumors [24]. This condition is one of the key factors limiting the efficacy of PDT [25]. In addition, PDT, which consumes oxygen, will further worsen tumor hypoxia, inducing stabilization of the hypoxia inducible factor, which triggers a cascade of signals that simultaneously promote the formation of new vessels, increase the invasive capacity of cells and facilitate their metastasis [26,27]. The problem of hypoxia in the tumor microenvironment can be alleviated by the use of precisely designed nanoparticles [28]. HSA/CAT-PEPA consists of human serum albumin, catalase, polymer(ethylpropylamino)ethyl methacrylate and the photosensitizer Chlorin e6 [23]. Human serum albumin is a very stable protein, resistant to pH changes, as well as being biodegradable and nontoxic, making it an ideal carrier for drugs. This albumin is naturally present in the human body, which reduces the risk of immunogenicity and toxicity. Additionally, it exhibits a long half-life (~19 days), which favorably influences the pharmacokinetics of bound drugs [29]. Furthermore, human serum albumin has the ability to accumulate in tumor tissues [30], and this effect is attributed to the effective interaction of albumin with the gp60 receptor, a vascular endothelial membrane protein, and SPARC, an extracellular matrix glycoprotein that is overexpressed in various tumor types and plays a key role in albumin transcytosis and promotes the local concentration of albumin-based nanocarriers loaded with therapeutic agent in tumors [31,32]. This ability may be particularly important in the case of Chlorin e6, used in the NP described here, which has a strong tendency to aggregate in physiological environments, reducing its performance as a photosensitizer and yielding poor pharmacokinetic and pharmacodynamic properties. Chlorin e6′s interaction with human serum albumin regulates its biodistribution, which may improve its targeting of cancer cells [33]. Importantly, human serum albumin reduces the immunogenicity of catalase and enables its stability in the blood circulation [23]. Catalase is an enzyme that breaks down hydrogen peroxide (H_2_O_2_) into water and oxygen, playing an important role in the reversal of hypoxia in cancer. The use of catalase in nanoparticles not only enhances the effect of PDT [34] but also enables the enhancement of the antitumor immune response [35], which is particularly attractive in the context of using PDT as a form of immunotherapy [36,37]. Polymer(ethylpropylamino)ethyl methacrylate is an ultra-acid-sensitive polymer that reacts to the slightly acidic tumor microenvironment, ensuring the stability of the nanoparticles in the circulation (neutral pH) and their dissociation in the tumor (acidic pH), allowing their activation at the appropriate site [23]. Chlorin e6 is a clinically approved photosensitizer [38], which represents a promising agent in anticancer nanomedicine based on photodynamic therapy. Nanoparticles containing this photosensitizer have achieved promising results in numerous cancer treatment studies [39]. Moreover, Chlorin e6 can enhance the immune response by inhibiting the PD-1/PD-L1 checkpoint [40]. Huang et al. tested HSA/CAT-PEPA NPs both in vitro on MDA-MB-231 triple-negative breast cancer cells and in vivo in a tumor model formed from these cells implanted subcutaneously into mice. In vitro, HSA/CAT-PEPA effectively reversed hypoxia. While, under hypoxia, cells treated with free catalase showed no improvement, the application of HSA/CAT-PEPA reduced the hypoxia rate by approximately 80%. Under hypoxia, ROS levels in cells treated with HSA/CAT-PEPA increased by more than 200% compared to the control group and by 50% more than with PS Ce6 alone. Under PDT with NP, tumor cell viability under hypoxia decreased to 20%, while, for Chlorin e6 alone, it was about 50%. HSA/CAT-PEPA showed significantly better penetration inside spheroids at pH 6.5 (80% deeper penetration compared to pH 7.4), indicating their high sensitivity to TME-specific acidic conditions [23]. However, it is worth noting that the MTT assay used in this study best detects rapid cell death (e.g., apoptosis), and slower mechanisms such as autophagy may lead to an underestimation of PDT efficacy in this assay [41]. In vivo application of HSA/CAT-PEPA increased oxygen saturation (SO_2_) in tumors by 60% compared to the control group. After the application of HSA/CAT-PEPA-PDT, tumor growth was inhibited by 85% compared to the control group without PDT and 50% more effectively than in the free Chlorin e6 group [23]. In conclusion, HSA/CAT-PEPA effectively reverses hypoxia, increases ROS generation and improves the efficacy of photodynamic therapy, both in vitro and in vivo, leading to significant inhibition of tumor growth and improved oxygen saturation (Figure 1).

Li et al. have developed novel nanoparticles targeting mitochondria with EHMONs-Ce6-CTPP@PFC [42]. Targeted effects on mitochondria during PDT lead to the release of cytochrome c, which acts as a key signal to initiate apoptosis [43]. The role of anticancer therapies targeting mitochondria in PDT has proven more effective than other similar nontargeted techniques. Especially in PDT, sensitizers targeting mitochondria are important because they play a key role in overcoming the hypoxia factor, resulting in high efficacy [44]. These NPs are constructed from eccentrically hollow mesoporous organic silica nanoparticles (EHMONs), the photosensitizer Chlorin e6, triphenylphosphine and perfluorocarbon compounds. Mesoporous organic silica nanoparticles (EHMONs) have a unique eccentric hollow structure, uniform size, large cavity and ordered mesoporous channels to construct nanoplatforms [42]. Chlorin e6 is the photosensitizer described earlier. Triphenylphosphine is a compound that targets mitochondria [45,46]. Importantly, triphenylphosphine is positively charged, which facilitates transport of the nanoparticle across the negatively charged mitochondrial membrane [47,48]. Perfluorocarbons are oxygen-carrying compounds. This allows EHMONs-Ce6-CTPP@PFCs to deliver oxygen to cancer cells, reducing hypoxia and increasing the effectiveness of PDT. EHMONs-Ce6-CTPP@PFCs were tested in vivo in a 4T1 mouse model of breast cancer. The nanoparticles significantly inhibited tumor growth (392 mm^3^) compared to the control group (1980 mm^3^). The stable body weight of the mice confirms the absence of acute toxicity [42].

Kang et al. developed a nanoparticle that is a porphyrin derivative, named TPP-O-PEG5 [49]. Porphyrins are among the most commonly used photosensitizers in PDT, and their unique properties—red fluorescence and preferential accumulation in cancer cells—make them promising for clinical applications [50]. TPP-O-PEG5 is a porphyrin modified by the introduction of four polyethyleneglycol-substituted phenyl groups at the meso positions of porphyrins. This structures hydrophilic polyethyleneglycol groups that readily bind water, which is important for the therapeutic properties of the nanoparticles [49]. Drug modification with polyethyleneglycol is a well-known technology for improving the physicochemical properties and biological response of a drug [51]. However, it is worth noting that patients may develop anti-polyethyleneglycol antibodies, which may limit the therapeutic efficacy of polyethyleneglycolylated substances as a result of inducing rapid clearance and neutralizing the biological activity of the substance [52]. During PDT, photoinduced charge transfer from the ether groups to the porphyrin ring occurs in the TPP-O-PEG5 structure, allowing the generation of reactive oxygen species and protons [49]. The generation of protons can enhance the effect of PDT and lead to cancer cell death [53]. Importantly, the proton production mechanism does not require the presence of oxygen, which enhances the efficacy of TPP-O-PEG5 under hypoxia [49]. TPP-O-PEG5 was tested both in vitro in breast cancer cell lines 4T1 and MDA-MB-231 and cervical cancer cell line HeLa and in vivo in a mouse model of breast cancer 4T1 and MDA-MB-231. In vivo, therapy with TPP-O-PEG5 effectively inhibited tumor growth in mice, particularly in large and highly hypoxic tumors, where tumor volume reduction reached approximately 80%. Approximately 60% of TPP-O-PEG5-treated mice achieved complete healing without recurrence within 30 days. The therapy was safe and no organ damage or other side effects were observed [49]. Although the overall results of the TPP-O-PEG5 NPs were promising, two main potential limitations associated with the research results can be noted. First, the quantum yield of TPP-O-PEG5 singlet oxygen in aqueous solution is only 0.22, while, in toluene, it is much higher at 0.61. Thus, it can be assumed that the efficiency of TPP-O-PEG5-PDT in an organism, where the environment is aqueous, may be lower. Second, in in vivo tests for smaller tumors (7.0–8.0 mm diameter), there was no significant difference in efficacy between TPP-O-PEG5-PDT and clinically accepted Talaporfin sodium-PDT, which may indicate that TPP-O-PEG5-PDT may not have a clear benefit in tumors that are not highly hypoxic.

**Figure 2:**
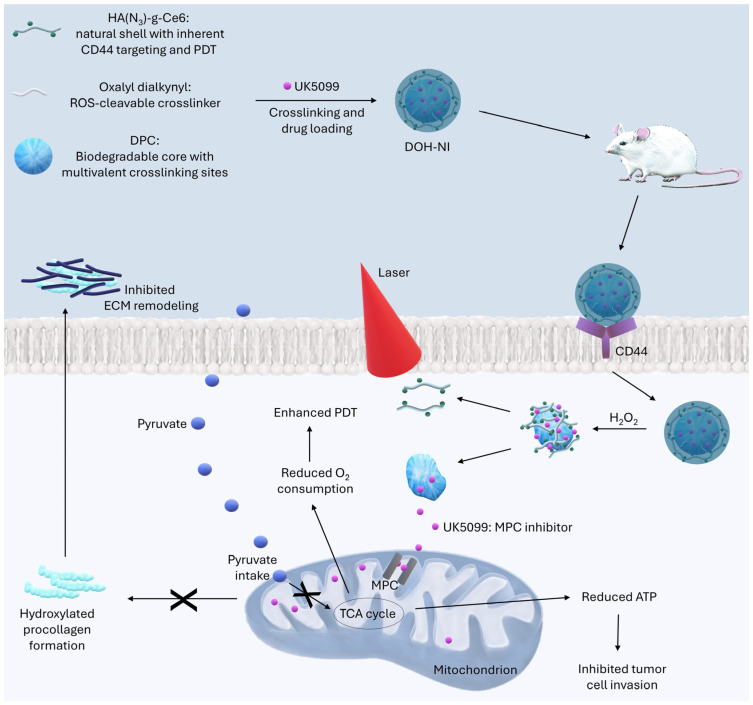
The figure shows the structure of the DOH-NI nanoparticle and its multidirectional mechanism of action. DOH-NI binds to the CD44 receptor, which is overexpressed in many cancer cells, enabling targeted endocytosis of DOH-NI by cancer cells. Once DOH-NI is internalized into tumor cells, its structure breaks down in response to the presence of ROS. This process results in the release of UK5099, an inhibitor of the mitochondrial pyruvate transporter (MPC). The MPC inhibitor reduces the transport of pyruvate into the mitochondria. This results in a decrease in ATP production due to inhibition of the TCA cycle (Krebs cycle), leading to a decrease in the energy available to cancer cells, inhibiting their invasiveness. A decrease in α-ketoglutarate (α-KG) levels, which prevents collagen hydroxylation and extracellular matrix (ECM) remodeling. This results in inhibition of premetastatic lung niche formation. The released photosensitizer Ce6 in the DOH-NI envelope enables the generation of ROS in response to illumination with 660 nm light. Inhibition of pyruvate metabolism by the MPC inhibitor reduces oxygen consumption by tumor cells, making more oxygen available for ROS during PDT. This, in turn, enhances the efficiency of cancer cell destruction [54].

Lv et al. constructed a DOH-NI nanoparticle possessing a core–shell dendritic structure designed to function in reactive TME, enabling efficient delivery and activation of the photosensitizer under specific tumor pathological conditions. The core of the nanoparticle consists of a biodegradable dendritic poly(carbonate) that contains hydroxyl and nitrogen groups. The structure of the core allows loading of the mitochondrial pyruvate carrier inhibitor, UK5099 [54]. The mitochondrial pyruvate carrier is a transport protein located in the inner membrane of mitochondria, responsible for the transport of pyruvate from the cytoplasm to the interior of the mitochondrion [55]. Due to its central metabolic role, inhibition of this protein may exhibit anticancer effects [56]. The nanoparticle coating, composed of hyaluronic acid, provides specific targeting to cancer cells that overexpress the CD44 receptor [54]. Furthermore, as a natural polysaccharide [57], hyaluronic acid has high hydrophilicity, excellent biocompatibility and nontoxicity [58,59,60]. Hyaluronic acid can also be degraded by enzymes that are overexpressed in the tumor environment, leading to the release of therapeutic cargo [58]. Chlorin e6, previously described, has been used as a photosensitizer. The oxalate bond between the core and the shell allows a response to changes in the intracellular environment, resulting in the release of the mitochondrial pyruvate carrier inhibitor and the therapeutic activation of the nanoparticle. The efficacy of the DOH-NI nanoparticle was tested in vivo in a 4T1Luc mouse model of breast cancer with lung metastasis, with impressive results—an 89% reduction in primary tumor growth compared to the control group. DOH-NI reduced pyruvate uptake in lung metastases by 62%, resulting in reduced collagen hydroxylation and preventing the formation of the premetastatic niche [54], a favorable secondary microenvironment for subsequent metastases [61]. The DOH-NI-PDT group showed the lowest level of pulmonary metastasis with a small bioluminescent signal, demonstrating effective prevention of metastatic development [54] (Figure 2).

Yan et al. developed polydopamine-based nanoparticles loaded with the photosensitizer curcumin [62]. Polydopamine has a rich chemistry, which allows post-functionalization of coatings using nanoparticles, polymers and proteins. Thanks to its properties, this material can function not only as a coating but also form nanoparticles, membranes and composites that interact with biomolecules and living cells [63]. Curcumin is a natural photosensitizer that usually occurs in the form of ketones and enols and is isolated from the rhizome of *Curcuma longa* [64]. Currently, this photosensitizer is in clinical trials for photodynamic antimicrobial and dental treatment [65]. Since curcumin is a very hydrophobic molecule, it requires some kind of formulation carrier to enable its use as a PS [66]. In PDA-Cur NP, curcumin is adsorbed on the surface of polydopamine through π-π and electrostatic interactions. This process allows for stable deposition of curcumin on the nanoparticles, which increases its durability and reduces the rate of photodegradation. Moreover, thanks to polydopamine, curcumin is less susceptible to photodegradation (by 46% under red light and by 50% under blue light). The antitumor activity of PDA-Cur nanoparticles was tested against mouse models of MCF-7 tumors implanted into Kunming mice. At day 8 after treatment, tumor volume after PDA-Cur-PDT decreased 2.03-fold relative to the saline-treated control group and was more effective than Cur-PDT, which showed a 1.34-fold decrease. As in other study groups, mice treated with PDA-Cur-PDT showed increased body weight after treatment, indicating that PDA-Cur NPs-mediated therapy had no significant side effects [62]. Although PDA-Cur NPs significantly improve the stability of curcumin, their ability to release the active ingredient over the long term may be limited. The NPs released 27.7% of curcumin in the first 2 h, and 41.9% was released after 8 h, suggesting that curcumin may be released rapidly in a lower pH environment [62]. The rapid release of curcumin means that its concentration at the site of action could quickly drop below effective levels, which could weaken the long-term photodynamic effect. In conclusion, although PDA-Cur NPs improve the stability of curcumin, their release mechanism may need further optimization to ensure a longer therapeutic effect. Moreover, the 450 nm light used in this work is strongly absorbed by tissue chromophores such as hemoglobin and melanin, resulting in very limited penetration—mainly into very shallow tissue layers, suitable for surface therapies [67].

Shen et al. have developed new photosensitizers whose emission arises from their aggregation based on pyridine-substituted triphenylamine salts with different alkyl chain lengths (TTP). Importantly, some of the synthesized photosensitizers can combine with albumin to form nanoparticles [68]. Unlike conventional fluorophores, which experience aggregation-induced quenching effects, aggregation-induced emission fluorophores show minimal emission in a good solvent but emit strong fluorescence in the aggregate due to a largely suppressed heat-scattering pathway through reduced intramolecular motion [69]. TTP nanoparticles respond to pH, allowing increased accumulation or endocytosis of the tumor, greatly facilitate mitochondria targeting, and have a strong ability to generate ROS. After double injection and irradiation 4 h after injection, TTP-treated 4T1 tumor mice showed a 92% decrease in volume, while the control group showed rapid growth [68].

**Figure 3:**
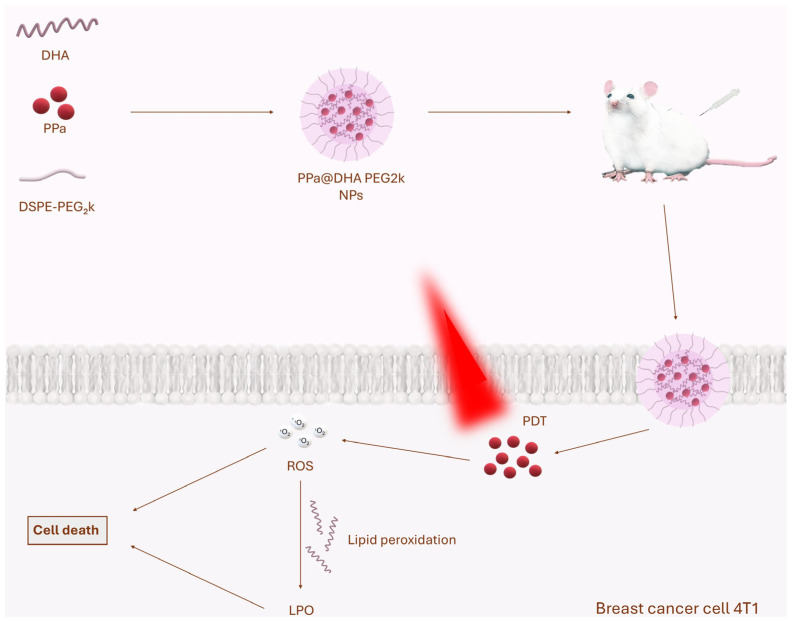
The figure shown illustrates the mechanism of action of PPa@DHA PEG_2_k nanoparticles in cancer therapy. These nanocomplexes consist of the photosensitizer Pyropheophorbide-a (PPa), docosahexaenoic acid (DHA) and the stabilizer DSPE-PEG_2k_. After intravenous administration in Balb/c mice with implanted 4T1 breast cancer, the nanoparticles are taken up by tumor cells via endocytosis. Upon exposure to laser light (λ = 660 nm), PPa is excited, leading to the generation of ROS, including singlet oxygen (^1^O_2_). The resulting ROS immediately induce damage to cellular structures. In addition, the presence of DHA in the nanoparticle structure enhances the therapeutic effect by intensifying lipid peroxidation. This process results in the formation of lipid peroxides (LPO), which have a longer half-life compared to ROS, resulting in a prolonged cytotoxic effect and enhanced destruction of tumor cell membranes. The synergistic effect of PDT and LPO leads to enhanced induction of cell death in a tumor model [70]. Han et al. developed novel CLIP-RB-PFOB@UCNP nanoparticles consisting of rose bengal photosensitizer and nanoliposome-based upconversion nanoparticles modified by CREKA peptide for PDT of NIR-triggered triple-negative breast cancer [79]. Rose bengal is an anionic, water-soluble xanthene dye and halogen derivative of fluorescein, which is a type II photosensitizer [80]. The compound is approved by the FDA as a dye for evaluating the ocular surface [81]. Tissue irradiation during PDT with these NPs can take advantage of well penetrating near-infrared wavelengths [82]. Upconversion nanoparticles are an important type of material for producing upconversion luminescence with an NIR laser, which stimulates them to efficiently emit UV/vis light [83]. Cys-Arg-Glu-Lys-Ala (CREKA) peptide is a desirable targeting ligand and has good ability to target fibrin overexpression in tumors [84]. The peptide has previously been successfully tested in various nanoparticles in triple-negative breast cancer models [85,86]. CLIP-RB-PFOB@UCNP NPs also contain perfluorooctane, which acts as an oxygen store, preventing tumor hypoxia and increasing the effectiveness of PDT. PDT with CLIP-RB-PFOB@UCNP was tested in a metastatic model of triple-negative breast cancer, where it effectively targeted and accumulated in tumor tissue thanks to CREKA and fibronectin, which is overexpressed in tumor cells. Under NIR irradiation, CLIP-RB-PFOB@UCNP showed significant destruction of orthotopic tumors, reduced hypoxia-inducible factor 1α levels and effectively inhibited lung metastasis [79].

Wang et al. developed novel PPa@DHA PEG_2k_ nanoparticles containing the photosensitizer Pyropheophorbide-a, docosahexaenoic acid and the polyethylene glycol modifier DSPE-PEG_2k_ [70]. Pyropheophorbide-a is a chlorine-based photosensitizer characterized by photostability and good absorption properties in the blue and red light regions [71]. Pyropheophorbide-a exhibits significant lipophilicity and a planar conformation, making it susceptible to aggregation in aqueous environments [72], in view of which nanotechnology approaches have been used to improve its delivery to tumors [73,74,75,76]. Polyunsaturated fatty acids have more double bonds and are easily oxidized to lipid peroxides under oxidation conditions [77]. Compared to singlet oxygen, lipid peroxides as lipid ROS have a longer half-life, which may increase the extent and duration of their action on cancer cells [78]. DSPE-PEG_2k_ is an FDA-approved stabilizing agent that enhances the stability of PPa@DHA PEG_2k_. Upon laser irradiation, the singlet oxygen produced by Pyropheophorbide-a rapidly oxidized docosahexaenoic acid, resulting in the formation of cytotoxic lipid peroxides. PPa@DHA PEG_2k_ was tested in vivo on a 4T1 mouse model of breast tumor. PPa@DHA PEG_2k_ nanoparticles + PDT reduced tumor volume by about 80%. Moreover, no adverse side effects were observed [70] (Figure 3).

Chen et al. developed novel photosensitizers self-assembled into PS-02 nanoparticles for effective type I PDT [87]. In type I PDT process, O_2_−, ·OH or H_2_O_2_ are produced by photoreactions based on electron transfer or hydrogen abstraction [88]. Type I PDT has shown significant potential in targeting hypoxic cancers due to its reduced O2 demand mechanism relative to type II PDT [89]. The core of the nanoparticle is a photosensitizer with heat-induced delayed fluorescence properties, to which piperazine was attached to increase electron transfer and enhance the type I reaction [87]. The photosensitizer is accompanied by a ligand 6-NS [87], which has the ability to not only target the breast cancer tumor cell marker carbonic anhydrase IX [90,91,92] but also regulating the electron transfer process for type I PDT. 6-NS formed a so-called “electron cage” that initially inhibited electron transfer, which resulted in blocking PDT, but, after binding to CAIX in the tumor microenvironment, the “cage” effect was unblocked, activating electron transfer and PDT. Amphiphil PS-02 tended to self-organize into PS-02 NPs, which exhibited a local “electron transfer cage effect” due to the electron-poor nature of 6-NS ligand. PS-02 nanoparticles were tested in vivo in the MDA-MB-231 triple-negative breast cancer model in mice. After light irradiation, the PS-02 NPs + light group showed significant tumor growth suppression, confirmed by histological analysis, with no noticeable side effects such as weight loss or damage to other organs. Tumor volume in the PS-02 NPs + light group decreased by approximately 70–80% compared to the control group [87]. It should be noted, however, that the light used in this study, with a wavelength of 532 nm, undergoes strong scattering and absorption in tissues, resulting in a limited depth of penetration—sufficient mainly for the treatment of superficial tumor lesions [67].

Zhuang et al. developed novel TQ@MOF-1 electron-transfer nanoparticles to produce type I hypoxia-resistant metal–organic structures by encapsulating thymoquinone [93]. Porphyrin-based nanoscale metal–organic structures have emerged as promising nanophotosensitizers for PDT. Their design depends on co-ordination interactions between porphyrin-based photosensitizers and metal ions/clusters, allowing high capacitance of the photosensitizers while ensuring their isolation to prevent self-quenching. In addition, the adjustable size and porosity of metal–organic structures facilitate accumulation at tumor sites and diffusion of ROS [94]. Thymoquinone, the main active ingredient in *Nigella sativa*, has excellent therapeutic properties in numerous in vivo and in vitro models. Nevertheless, the molecule is not yet in clinical trials, mainly due to its poor bioavailability and hydrophobicity [95]. Previous studies have shown that thymoquinone can also act as an effective mediator of electron transfer [96]. The present study confirms this concept, showing that it facilitates electron transfer from the photosensitizer ligand embedded in the metal–organic backbone to oxygen, which promotes activation of the type I pathway while weakening the primary type II mechanism. NP TQ@MOF-1 was tested under in vivo conditions in a 4T1 mouse model of breast cancer. These NPs exhibit enhanced antitumor activity under hypoxic conditions and superior in vivo antitumor efficacy compared to native MOF-1 NPs. TQ@MOF-1 NPs show significant therapeutic potential in tumor PDT, effectively inhibiting tumor growth while maintaining good biocompatibility. The observed decrease in tumor volume in the TQ@MOF-1 NPs + light group was about 85% compared to the control group after 21 days [93].

Cui et al. developed a novel PSe nanoplatform (POEGMA-b-P(PSeMA-co-TPPC6MA)), designed as a self-adaptive photosensitizing carrier that activates in the tumor microenvironment to increase the precision of tumor targeting. PSe consists of hydrophilic segments of POEGMA (poly(oligoethylene glycol) methacrylate), selenium units in the form of an alkyl aryl selenide (PSeMA) and hydrophobic segments containing the photosensitizer, porphyrin TPPC6MA [97]. POEGMA are amphiphilic polymers that have a hydrophobic main chain and hydrophilic side chains based on oligo(ethyleneglycol). These polymers have well-documented bio-inert and nontoxic properties, as they do not exhibit specific interactions with biological materials [98,99,100] and also do not induce an immune response, are not recognized by poly(ethylene glycol) antibodies and do not induce the production of anti-POEGMA antibodies [101,102]. For PSe nanoparticles, POEGMA contributes to high biocompatibility and stability in the bloodstream. The selenide units used embedded in the structure of the nanoplatform reduce the aggregation of porphyrins, which, in turn, leads to a higher production of singlet oxygen. The PSe nanoparticles were tested in vivo in a mouse model of 4T1 breast cancer. Mice treated with PDT with PSe nanoparticles showed a tumor volume reduction of more than 70% within 14 days, compared to the control group [97].

The nanoparticles described in the Zhang et al. study are chemically modified perylenediimides (PDIs), referred to as TBDT(TriBromo-Dipyrrolidine-Tethered). These nanoparticles contain three bromine atoms and one pyrrolidine group at the “bay” position of the PDI core. The introduction of heavy bromine atoms reduces the energy difference between the ground and excited states (ΔEST), promoting the inter-system transition (ISC) process. The structure of these molecules is based on the donor-π-acceptor (D-π-A) principle, which enhances their ability to generate ROS when exposed to near-infrared light [103], which has good tissue penetration [82]. An in vivo study used a 4T1 mouse model of breast cancer. After 14 days, the therapy reduced tumor volume by ~90% compared to the control groups. TBDT-PDT significantly increased the activation of CD8+ T lymphocytes and dendritic cells in the tumor and decreased M2 macrophage levels [103].

**Figure 4:**
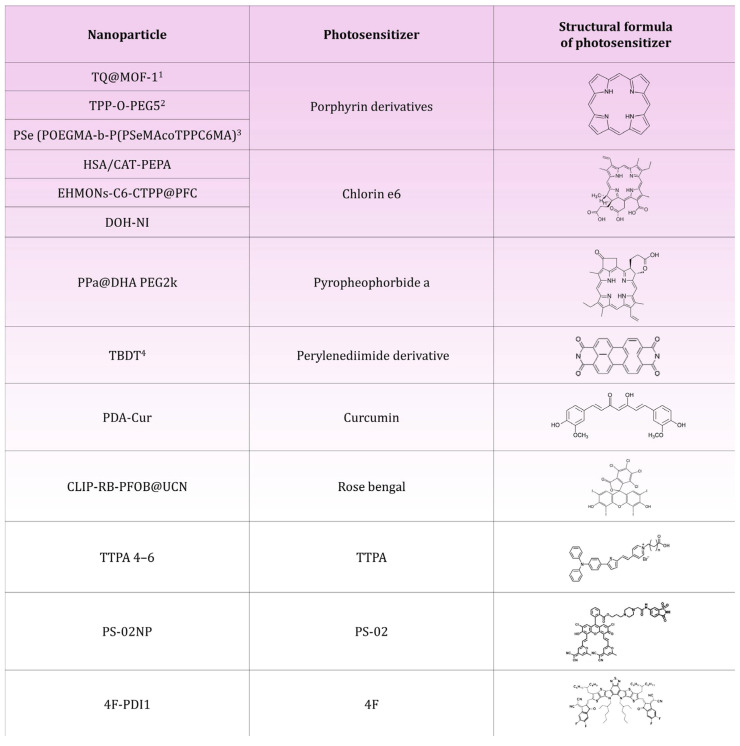
The figure shows a table listing the nanoparticles described in the article along with their respective photosensitizers and their chemical structures. 1. TCPP-Tetrakis(4-carboxylphenyl)-porphyrin; 2. TPP-O-PEG5-porphyrin modified by the introduction of four polyethyleneglycol (PEG)-substituted phenyl groups at the porphyrin mesoparticle positions; 3. TPPC6MA-tetraphenylporphyrin modified with a six-carbon chain; 4. TBDT-perylenediimide modified with three bromine (Br) atoms in the bay position and a pyrrolidine group.

Hu et al. have developed a 4F-PDI1 nanoparticle that utilizes a mechanism to enhance the separation of electron–hole pairs via photoinduced electron transfer. The key element of the nanoparticle structure is the L8-BO-EH-4F (4F) compound, based on a semiconductor electron backbone. The core of the molecule contains sulfur atoms that enhance the donor properties and facilitate charge transfer, while the ends of the molecule have been modified with fluorinated dicyanovinyl groups acting as electron acceptors [104]. Another component of the nanoparticle is perylene diimide, a photoactive compound with electron donor properties [105]. The stability and integrity of the nanoparticle is provided by the polymer Pluronic F-127, which, as an amphiphilic stabilizer [106], promotes the formation and stability of the system [104]. 4F-PDI1 was tested in vivo in the 4T1 mouse model of breast cancer. In these mice, a more than 80% reduction in tumor volume was observed within 16 days [104] (Figure 4, Table 2).

## 3. Conclusions

Due to the complex biological heterogeneity of breast cancer and the need to individualize the therapeutic approach depending on its subtype, the search for new, more effective treatments remains a priority for modern oncology. Breast cancer is currently the most frequently diagnosed malignancy worldwide and the leading cause of cancer deaths among women. Moreover, the observed annual increase in incidence indicates an urgent need to develop innovative therapeutic strategies that could improve the prognosis and quality of life of patients. Nanoparticles in PDT represent an innovative approach to breast cancer treatment, offering significant improvements in stability, bioavailability and selective delivery of photosensitizers. Nanotechnology-assisted PDT has shown promising results in preclinical studies, indicating an increase in therapeutic efficacy and beneficial effects on the tumor immune microenvironment. However, despite these advances, the clinical application of PDT still faces significant challenges. One of the main limitations of PDT is tumor hypoxia, as the efficacy of this method is strictly dependent on oxygen availability. Tumors, including breast cancer, are often characterized by areas of significant hypoxia, which limits the effectiveness of ROS generation. In response to this challenge, novel nanoparticles are being developed to increase oxygen availability in the tumor microenvironment, for example, by using perfluorocarbonates as oxygen carriers or using catalase to convert hydrogen peroxide to oxygen. Another major problem is the limited penetration of light in biological tissues, which significantly reduces the effectiveness of PDT for tumors located in deeper layers. In this context, the use of up-conversion nanoparticles capable of converting near-infrared radiation into visible light is a promising strategy to improve the therapeutic efficacy of PDT through deeper light penetration. Optimizing the delivery and activation of photosensitizers is also a key area of research. Nanoparticles can be designed to selectively accumulate in tumor cells to minimize side effects in healthy tissues. This includes strategies based on tumor receptors, such as CD44 for targeted delivery of hyaluronic acid, and smart carrier systems that respond to specific physiological stimuli, including pH or enzymatic activity. PDT, as a topical therapy, has shown limited efficacy in treating cancers with metastasis to multiple organs. Although preliminary studies indicate that nanoparticles have the potential to enhance the efficacy of PDT in eliminating metastases, their effectiveness in this regard is still limited and requires further research. The long-term safety of nanoparticles in PDT remains a key issue requiring careful evaluation in clinical trials. Further monitoring of their biocompatibility, metabolism and potential side effects is needed. It is worth noting that all in vivo studies conducted in 2024 were conducted in mouse models in which the tumors are quite small. No clinical trials of the nanoparticles described have been conducted. To achieve breakthroughs in the treatment of breast cancer, further research into the interactions of nanoparticles with tumor cells and the tumor microenvironment, the development of technologies to improve light penetration, and the development of multifunctional therapeutic systems are needed. The integration of nanoparticles can significantly enhance the efficacy and safety of PDT, contributing to advances in cancer therapy.

## Figures and Tables

**Table 1:** Criteria for inclusion and exclusion of retrieved articles for overgrowth.

**Inclusion Criteria**
Articles describing photodynamic therapy
Articles describing cancer therapy
Articles describing nanoparticles
Articles published in 2024
**Exclusion criteria**
Articles describing photodynamic therapy combined with other forms of therapy (chemotherapy, radiation therapy, gene therapy, photothermal therapy, etc.) or imaging
Articles describing cancers other than breast cancer
Articles other than original research papers
Articles in which the results of therapy were described only in vitro
Articles in a language other than English and Polish

**Table 2:** Summary of nanoparticles described in the article.

Name of the Nanoparticle	Construction	Results	Quoting
HSA/CAT-PEPA@Ce6	Serum albumin (HSA), catalase (CAT), PEPA polymer, Chlorin e6	Reduction in MDA-MB-231 breast cancer volume by 90%	[23]
EHMONs-Ce6-CTPP@PFC	Eccentric hollow mesoporous organic silica nanoparticles (EHMONs), triphenylphosphine (CTPP), Chlorin e6, perfluorocarbons (PFCs)	Reduction in 4T1 breast cancer volume by 80%	[42]
TPP-O-PEG5	Modified porphyrin with four phenyl groups with a polyethyleneglycol (PEG) substituent	Reduction in breast cancer volume of 4T1 and MDA-MB-231o 73% for tumors with a diameter of 7.0–8.0 mm; reduction in breast cancer volume of 4T1 and MDA-MB-231o 89% for tumors with a diameter of 9.0–11.0 mm	[49]
DOH-NI	Biodegradable dendritic poly(carbonate) (DPC), mitochondrial pyruvate carrier (MPC) inhibitor UK5099, hyaluronic acid (HA) coating, Chlorin e6	Reduction in volume of cancer 4T1Luc breast by 89%; reduction in pyruvate uptake in pulmonary metastases by 62%	[54]
PDA-Cur	Polydopamine core (PDA), curcumin (Cur)	Reduction in MCF-7 breast cancer volume by 51%	[62]
TTPA 4–6	Triphenylamine (TPA), pyridine fragment, alkyl chains	4T1 breast cancer volume reduction of 85% for TTPA 4, 92% for TTPA 5 and 88% for TTPA 6	[68]
PPa@DHA PEG_2_k	Pyropheophorbide-a (PPa), docosahexaenoic acid (DHA), stabiliser DSPE-PEG_2_k	Reduction in 4T1 breast cancer volume by 80%	[70]
CLIP-RB-PFOB@UCNP	UCNP core, CLIP liposomal coating, Rose Bengal, Perfluorooctane (PFOB)	Significant reduction in breast cancer volume of TNBC model; reduction in HIF-1α levels; inhibition of lung metastasis	[79]
PS-02	PS-02 thermally activated delayed fluorescence photosensitizer (TADF), piperazine, 6-NS ligand	Reduction in MDA-MB-231 breast cancer volume by 80%	[87]
TQ@MOF-1	MOF-1 core (PCN-224), TCPP ligand, thymoquinone (TQ), F-127 coating	Reduction in 4T1 breast cancer volume by 85%	[93]
PSe (POEGMA-b-P(PSeMA-co-TPPC6MA)	POEGMA (Poly(oligoethylene glycol) methacrylate), selenium units in the form of selenide (PSeMA), tetraphenylporphyrin (TPP) modified with a methacrylate-terminated hexyl side chain (TPPC6MA)	Reduction in 4T1 breast cancer volume by 70%	[97]
TBDT	Perylenediimide (PDI) with bromine and pyrrolidine	Reduction in 4T1 breast cancer volume by 90%; increase in activated CD8+ T lymphocytes, dendritic cells and decrease in M2-type macrophages	[103]
4F-PDI1	L8-BO-EH-4F semiconductor, perylenodiimide (PDI), Pluronic F-127	Reduction in 4T1 breast cancer volume by 80%	[104]

## Data Availability

No new data were created or analyzed in this study. Data sharing is not applicable to this article.
